# Development and validation of the Manipal Interstitial Lung Disease Education Booklet (MILD EduB) for individuals with interstitial lung disease

**DOI:** 10.1371/journal.pone.0353274

**Published:** 2026-07-14

**Authors:** Revati Amin, Aswini Kumar Mohapatra, G. Arun Maiya, Vishak Acharya, Shivashankara Kaniyoor Nagri, Suresh Sukumar, Winniecia Dkhar, Anup Bhat, Mukesh Kumar Sinha, Marita T. Dale, Jennifer A. Alison, K. Vaishali

**Affiliations:** 1 Department of Physiotherapy, Kasturba Medical College Mangalore, Manipal Academy of Higher Education, Manipal, India; 2 Department of Respiratory Medicine, Kasturba Medical College, Manipal Academy of Higher Education, Manipal, India; 3 Department of Physiotherapy, Manipal College of Health Professions, Manipal Academy of Higher Education, Manipal, India; 4 Department of Pulmonary Medicine, Kasturba Medical College Mangalore, Manipal Academy of Higher Education, Manipal, India; 5 Department of Medicine, Kasturba Medical College, Manipal Academy of Higher Education, Manipal, India; 6 Department of Medical Imaging Technology, Manipal College of Health Professions, Manipal Academy of Higher Education, Manipal, India; 7 Sydney School of Health Sciences, Faculty of Medicine and Health, The University of Sydney, Sydney, Australia; 8 Allied Health, Sydney Local Health District, Sydney, Australia; The University of Kansas Health Systems St. Francis Campus, UNITED STATES OF AMERICA

## Abstract

In India, Interstitial Lung Disease (ILD) is a significant healthcare burden. Poor outcomes are caused by delayed diagnosis and insufficient patient education (PE). In this context, the Manipal Interstitial Lung Disease Educational Booklet (MILD EduB), a culturally relevant resource for individuals with ILD in India, was developed and validated. The booklet was developed in four stages: (1) a scoping review to identify important educational themes; (2) development of a draft booklet shared with experts for review and feedback; (3) validation of the booklet by healthcare professionals using the content validity index (CVI); and (4) face validation by individuals with ILD. The initial scale-level CVI (S-CVI/Ave) was 0.79, which improved to 1.0 following modifications, indicating good expert consensus via the healthcare professionals. Face validation by individuals with ILD (n = 10) using a 4-point Likert scale demonstrated 100% agreement for readability, relevance, and design. With a Flesch Reading Ease Scale score of 80.9, the finalised booklet is considered easy to read and accessible to individuals with ILD with a wide range of reading levels. MILD EduB fills an important gap in ILD-specific educational resources, particularly in low-resource settings. Its evidence-based content and patient-centered design aim to improve adherence and self-management.

## Introduction

Interstitial lung disease (ILD) is a collection of more than 200 chronic respiratory diseases characterized by inflammation and fibrosis of the lung interstitium, resulting in reduced gas exchange and progressive respiratory deterioration [1]. The disease progresses at various rates, ranging from slow to rapidly deteriorating forms, with idiopathic pulmonary fibrosis (IPF) being one of the most severe subtypes [2]. The prevalence of ILD ranges from 7 to 1650 per 100,000 people [3], resulting in a significant worldwide health burden. With a median survival of 2–5 years after diagnosis, the prognosis remains poor [2].

The epidemiology of ILD in India differs from that in Western countries, with a higher prevalence of hypersensitivity pneumonitis (HP), sarcoidosis, connective tissue disease-associated ILD (CTD-associated ILD), and IPF, which are most likely due to genetic, occupational, and environmental factors [4,5]. Exposure to industrial pollution, agricultural dust, and biomass fuels increases the disease burden, necessitating regionally customized management techniques [6,7]. ILD symptoms include fatigue, persistent dry cough, and progressive dyspnea [1]. Nonspecific presentations, which are typically caused by limited access to specialized care and are misdiagnosed as chronic bronchitis, asthma, or tuberculosis, occasionally result in diagnostic delays of 1.5–2 years [8–10].

Both pharmaceutical and nonpharmacological approaches are used for the management of ILD. Antifibrotic medications (e.g., pirfenidone and nintedanib) constitute the mainstay of treatment for IPF, whereas immunosuppressants (e.g., corticosteroids, mycophenolate) are used to treat inflammatory subtypes [11–14]. Pulmonary rehabilitation (PR), which involves exercise training and patient education both of which are essential for improving functional capacity and self-management, nutritional support, and psychological counseling, improves functional capacity and quality of life (QoL) [15]. Self-management techniques, such as smoking cessation and the avoidance of specific triggers, are essential for preventing the progression of the disease [16–20]. Healthcare professionals and patients equally appreciate the importance of these self-management educational initiatives involving self-management within PR since they provide people with the necessary skills to change behavior and maintain favorable health outcomes [18,19]. However, long-term adherence to beneficial health behaviours remains challenging in the absence of structured patient education. The information given to patients should be explained in an accessible manner. Printed educational materials have been used to encourage patients to commit to taking care of themselves and to increase comprehension, feeling of well-being contentment, and treatment adherence [21–25]. Furthermore, it is recommended to reinforce written educational resources designed by medical professionals using verbal communication [26].

Healthcare professionals utilize educational interventions for imparting knowledge and evaluating health education resources [26]. Through interactions mediated between patients, family members, and healthcare professionals, the increasing prevalence of written and visual educational materials facilitates the teaching and learning process [21–26]. However, in India, the lack of standardized, culturally relevant educational tools exacerbates health literacy gaps, with patients frequently depending on unverified online sources or informal guidance [27]. Currently, educational materials for ILD patients have been sparse in India and worldwide; many of the available resources focus on COPD. Many of the educational materials consist of hospital handouts, society brochures, and internet resources; however, most of them do not contain information unique to ILD and are generally targeted toward the literate population. The majority of available resources are either native English or based on Western guidelines, making them inaccessible to rural and non-English-speaking individuals in India. In addition, current resources are often generalized and do not address the unique challenges faced by ILD patients on an individual basis. To meet this need, MILD EduB (Manipal Interstitial Lung Disease Educational Booklet) was created to deliver ILD specific education in a culturally appropriate format to Indian patients. While current resources emphasize the role of exercise as a component of PR, none of the previous educational materials specifically emphasized the importance of regular exercise in the management of ILD. As well, unlike other educational materials, MILD EduB provides a culturally adapted version of an educational book for the needs of ILD patients based upon the social economic factors and health belief systems common in India. Additionally, the design of MILD EduB included consideration for the varying degrees of literacy of those who would use it; it contains clear, large print, and simple graphics to aid in comprehension for those with less than high school educations.

Additionally, MILD EduB addressed local dietary habits, potential triggers for ILD, and health beliefs that are prevalent in India. Moreover, a rigorous content validity process was completed using the Content Validity Index (CVI). Thus, the MILD EduB content received expert consensus on its relevance and accuracy. There is a critical need to develop and validate an educational booklet about exercise, specifically designed for individuals with ILD to promote safe and effective exercise at their place of residence. By creating a targeted educational booklet, we may provide patients with the necessary knowledge to encourage incorporation of exercise into their daily routine, potentially improving their physical and psychological health and long-term adherence.

Effective self-management and care optimization are hampered by the lack of a validated, culturally appropriate tool tailored for individuals with ILD, even though structured patient education has been shown to be effective in other chronic respiratory diseases (CRDs) [27–33]. However, there is currently no proven ILD-specific educational resource for the Indian community. ILD is still a major but underappreciated healthcare problem in India, where poor outcomes are caused by delayed diagnoses, restricted access to treatment, and inadequate availability of patient-centered educational materials [8–10]. Addressing this gap through a specifically designed educational booklet has the potential to empower patients, improve therapy adherence, and improve overall disease prognoses. In addressing a key void in ILD specific patient education in India, this Phase 1 study is to develop and validate a comprehensive educational resource for persons with ILD in India. Only the development, content validation, and face validation of the MILD EduB are presented in this manuscript. A future randomized controlled trial will evaluate the impact of the booklet on patient knowledge, compliance/adherence, self-management, and clinical outcomes. MILD EduB is a component of a larger RCT which combines educational content with home-based PR. It is expected that this combination will ultimately enhance patient outcomes by providing ILD patients with both information and motivation to adhere to an exercise program utilizing home based rehabilitation.

## Materials and methods

This study is a part of a previously designed randomized controlled trial (RCT) protocol that follows the Consolidated Standards of Reporting Trials (CONSORT) guidelines [34]. As described in the protocol, the outcomes of this study constitute Phase 1, i.e., the development and validation of an exercise educational booklet for individuals with ILD in India. All participants received a comprehensive participant information sheet detailing the study’s goals, methods, possible advantages, and risks prior to enrollment. Every participant provided written informed consent. The consent procedure made it clear that participation was voluntary and that their standard of medical care would not be impacted if they chose not to participate or later withdrew. The study protocol and consent forms were reviewed and granted approval by the Kasturba Medical College and Kasturba Hospital (KMC and KH) Institutional Ethics Committee, approval number- IEC: 320/2020, and the study was prospectively registered under the Clinical Trials Registry of India (CTRI) (CTRI/2020/09/027788) on 14/09/2020 [34]. The participants were recruited from 18 August 2020 to 29 December 2023 for the study.

The methodology for the development and validation of the educational booklet followed four stages

Stage 1: Scoping review to identify educational content for individuals with ILD

Stage 2: Development of the exercise educational booklet

Stage 3: Validation of the exercise educational booklet by healthcare professionals.

Stage 4: Evaluation of the exercise educational booklet by the targeted patient group.

The steps are sequentially described below:

Step 1: Scoping review to identify educational content for individuals with ILD.

PR was originally designed to assist individuals with chronic obstructive pulmonary disease (COPD) [35], which has different causes and symptoms than ILD. Individuals with other CRDs, such as ILD, may not benefit from COPD-specific education such as the basis for exercise limitations, treatment and disease outcomes. While Patient Education (PE) is frequently provided verbally to individuals with ILD by healthcare professionals, little is known regarding the precise content, mechanisms, and overall effectiveness of this education. The dearth of information regarding the recommended amount and content of PE for individuals with ILD necessitated a scoping review of the literature. As a first step in identifying the contents of our exercise educational booklet, we therefore published a scoping review to determine the major themes of PE content utilized to deliver education to individuals with an ILD [19]. This scoping review identified key patient education domains relevant to ILD, which were subsequently used to inform booklet content development.

Step 2: Development of the educational exercise booklet.

The educational booklet was methodically developed by RA and VK, with relevant detailed images, clear syntax, and important information from stage 1 of the phase 1 methodology. These components were included in an initial draft educational exercise booklet. A Flesch Reading Ease score for the final version of the MILD EduB booklet was obtained to ensure accessibility and readability. The booklet received a score of 80.9 demonstrating that it was easy to read and appropriate for a wide audience [36–38]. A panel of seven expert clinicians, comprising three pulmonologists and four cardiopulmonary physiotherapists, thoroughly reviewed the initial preliminary draft. The panel evaluated the content in terms of accuracy, comprehensiveness, and relevance to ensure that the booklet adhered to strict criteria for reliability and quality in cardiopulmonary health education and provided expert feedback through both a rating criteria and written suggestions [39].

Step 3: Validation of the educational exercise booklet by healthcare professionals.

Initial Draft Review: The Item-Level Content Validity Index (I-CVI) [39] was used in this comprehensive evaluation. Using a 4-point Likert scale (where 1 = very irrelevant and 4 = very relevant), each item in the booklet was evaluated by the same healthcare professionals for accuracy, comprehensiveness, relevance, and clarity. The health professionals provided thorough feedback via structured forms, commenting on each component of the booklet.

We calculated the I-CVI for each item using the following formula [39]:


$$\begin{array}{c} I-\text{CVI}=\frac{\text{Number\textasciitilde{}of\textasciitilde{}healthcare\textasciitilde{}professionals\textasciitilde{}rating\textasciitilde{}the\textasciitilde{}item\textasciitilde{}as\textasciitilde{}relevant\textasciitilde{}on\textasciitilde{}the\textasciitilde{}Likert\textasciitilde{}scale}}{\text{Total\textasciitilde{}number\textasciitilde{}of\textasciitilde{}healthcare\textasciitilde{}professionals}} \end{array}$$

Integrating feedback: All of the comments were combined into one large document and arranged on the basis of the components of the booklet they addressed. To help with integration, common themes and recommendations from expert healthcare professional feedback were identified. To resolve any conflicting feedback, the authors (RA and VK) engaged collaboratively in examining and editing the MILD EduB booklet according to the healthcare professional’s recommendations.

Revision process: The draft booklet was revised on the basis of the aggregated feedback and healthcare professional’s recommendations. A second validation round was conducted with a reduced panel of five healthcare professionals (Pulmonologists and Cardiopulmonary Physiotherapists) on the updated written material. By offering additional comments and verification, these experts ensured that the information fulfilled the requirements for accuracy, relevance, and utility.

The scale-level content validity index, i.e., S-CVI/Ave was calculated to provide additional evidence of overall content validity [39].

The average of the I-CVI scores for each item on the booklet is known as the S-CVI/Ave [39]. It was determined via the following formula, which offers a comprehensive assessment of the content validity of the entire booklet:


$$\begin{array}{c} S-\frac{CV\text{I}}{\text{Ave}}=\frac{\text{Sum\textasciitilde{}of\textasciitilde{}}I-\text{CVI\textasciitilde{}for\textasciitilde{}all\textasciitilde{}items}}{\text{Total\textasciitilde{}number\textasciitilde{}of\textasciitilde{}items}} \end{array}$$

This index evaluates the booklet’s overall validity as a unified entity, ensuring that all sections merge to provide complete and cohesive information. This thorough validation approach ensured that the booklet maintained a consistently high level of quality while also offering in-depth coverage of each topic item.

Step 4: Evaluation of the educational booklet by the targeted patient group.

To further validate the educational booklet, ten people with ILD aged 40–70 years were invited to participate in the evaluation process via convenience sampling from the Department of Respiratory Medicine, Kasturba Medical College, MAHE, Manipal. These patients provided valuable feedback on the booklet from the perspective of its intended audience.

Several important elements of the booklet were the focus of a careful recording of the participants’ percentage agreement. These factors included readability, design, layout, clarity, organization, visual aids, and content.

**Fig 1:**
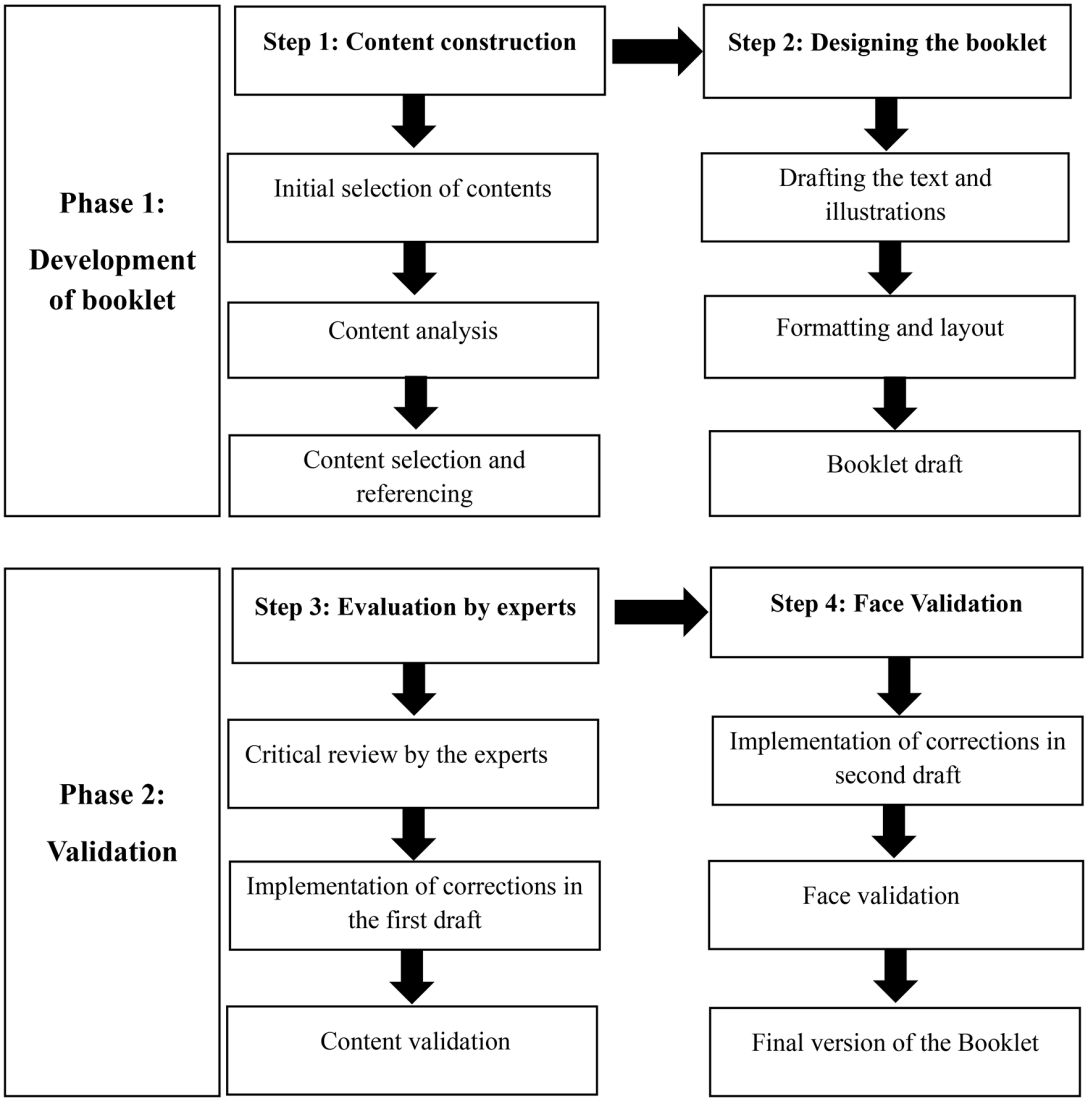
Pictorial demonstration of the methodology.

Following a careful examination of each participant’s input, the degree of agreement was measured via a 4-point Likert scale (where 1 = very irrelevant and 4 = very relevant). This thorough feedback ensured that the booklet was not only factual and educational but also appropriate for patients with varying degrees of health literacy to use and comprehend. (Fig 1)

### Statistical analysis

The CVI was used to validate the MILD EduB, which comprised both the I-CVI and S-CVI/Ave. The I-CVI was determined for each item of the booklet by calculating the proportion of experts who assessed the item as relevant on a 4-point Likert scale. The I-CVI was interpreted as per prior published recommendations. A minimum I-CVI value of 0.71 or greater was defined as acceptable for a panel of seven experts. Values of.80 or higher indicated even stronger agreement among the experts. Thus, items with an I-CVI value of 0.71 during Round 1 were retained and modified at the discretion of the experts to better reflect clarity, relevance, and comprehensiveness. The I-CVI scores for each item were averaged to determine the S-CVI/Ave, which represented the booklet’s overall validity. The CVI scores could range from −1–1, with higher values suggesting greater consensus among experts [39]. The booklet’s usability, design, and content had to be unanimously agreed upon by at least 70% of the individuals with an ILD to be considered clear and pertinent.

## Results

The study results will be presented in stages to enhance clarity in reporting the results. The following sections provide stepwise details on the results.

Step 1: Themes identified through the literature review (Scoping review): Thirteen articles were included in the scoping review, which were compiled, summarized, and results extracted. The results of the scoping review have been previously published [19] and were utilized as content (themes) for the development of the educational booklet.

Step 2: Development of an exercise educational booklet

The educational topics that were included in the exercise educational booklet were based on the results of the scoping review [19] and included: Pathophysiology of ILD, clinical tests for ILD, managing breathlessness/dyspnea, managing cough, managing flare-ups, prognosis, managing anxiety, panic, and depression, using supplemental oxygen, using nocturnal oxygen, regular vaccination, managing medications and side effects, good nutrition, managing coexisting medical conditions, accessing home-care and support for patients and caregivers, quitting smoking, symptoms of gastroesophageal reflux disorder, adherence to medications, life-style changes, exercise, end-of-life care and advanced directives, benefits of pulmonary rehabilitation, importance of exercise, and taking control of their own health. The organization of the content in the booklet utilized simple language, bullet points, and visuals that supported a thematic structure to allow the reader to quickly access information about symptoms, improving exercise performance, how much oxygen is being used, and monitoring oneself. The selections of the visual aids (supportive images) were intended to support the message of the section in regards to managing symptoms, exercise performance, oxygen use, and self-monitoring. The format design, including the size of the font, space between lines of type, and overall layout of the sections of the booklet were deliberately made to improve the ability of readers with differing levels of health literacy to read and understand the content of the booklet.

Step 3: Content validation by experts: The Content Validity Index (CVI) was used to quantify expert agreement on the relevance and comprehensiveness of each booklet item’s content.

According to the average method (S-CVI/Ave), the booklet’s overall S-CVI was 0.79, which indicates a high level of content validity. Ten items received a CVI of 0.71, whereas one item received a CVI of.89. Both indicate that there was strong agreement among the experts concerning relevance. Even though multiple items had an I-CVI value of 0.71 during Round 1, it was determined acceptable for a group of seven experts. The authors incorporated every comment provided by the experts concerning each item to further reinforce clarity, relevance, wording, and presentation for each item prior to continuing on to the second round of validation. Using the written suggestions and improvements were made to the second draft of the MILD EduB. The experts reviewed all the revised items, including the previously provided written comments. All the items in the booklet received an I-CVI of 1.0 in the second round of assessment, indicating that all the experts agreed on the relevance and appropriateness of the items. (Tables 1 and 2)

**Table 1:** Content validation by experts (I-CVI and S-CVI/Avg).

Sr no.	Items	I-CVI Round 1	I-CVI Round 2
1	Front cover		
	• Cover page	1	1
	• Title	1	1
2	Writing Skills		
	• Font style and size	0.71	1
	• Line spacing	0.71	1
3	Structure and presentation		
	• Arrangement of contents	0.71	1
	• Language is easy to understand	0.71	1
	• Illustrations are appropriate	0.71	1
	• Content socially and culturally appropriate	0.71	1
	• Suitable number of pages	0.71	1
4	Objectives		
	• Coincides with target population	1	1
	• Can be circulated within the community	0.71	1
	• Can improve knowledge, attitude and practice among ILD patients with respect to disease management	0.71	1
5	Relevance		
	• Quality of information	0.71	1
	• Sufficiently specific and comprehensive material	0.89	1
S-CVI/Avg	0.79	1

Abbreviations: I-CVI- Item-level content validity index, S-CVI/Avg: Scale-level content validity index, Sr. No.- Serial Number.

**Table 2:** Recommendations from healthcare professionals on the exercise educational booklet after round 1.

Sr. No	Recommendations from healthcare professionals
1	Consider putting Manipal on one line; Interstitial Lung Disease on one line; Educational booklet on one line.
2	Spell check- Psychologist. Font can be changed to more legible font.
3	When you say GERD – not sure if patients/caregivers would understand this-giving a brief explanation. Know your symptoms: -Delete ‘followed by’. -Consider splitting this sentence to 2: limitation of routine activities. And then in the next tab say symptoms usually progress rapidly. Triggers (AVOID): This heading is confusing. You could just write avoid. Also, instead of worry, consider replacing with stress. Try to get professional help in arranging the illustrations. Try searching literature, the triggering factors in this geographical area (coastal Karnataka),like fungus, molds.
4	Limited use to English speaking population. Talk about importance of monitoring during exercise – SpO2/ physiological parameters etc. May need some work to ensure that the general community understands the brochure.

Step 4: Face validation by ILD patients: The revised booklet received extremely positive feedback from participants with ILD. With respect to the participants’ views on the educational booklet, there was 100% agreement on all items (Table 3). This unanimous agreement emphasizes the usefulness and appropriateness of the booklet as an educational resource for people with ILD.

**Table 3:** Face validation by ILD patients.

Sr no.	Items	Number of participants (n = 10)	Percentage of agreement
1	Front cover		
	• Cover page	10	100%
	• Title	10	100%
2	Writing Skills		
	• Font style and size	10	100%
	• Line spacing	10	100%
3	Structure and presentation		
	• Arrangement of contents	10	100%
	• Language is easy to understand	10	100%
	• Illustrations are appropriate	10	100%
	• Content socially and culturally appropriate	10	100%
	• Suitable number of pages	10	100%
4	Objectives		
	• Coincides with target population	10	100%
	• Can be circulated within the community	10	100%
	• Can improve knowledge, attitude and practice among ILD patients with respect to disease management	10	100%
5	Relevance		
	• Quality of information	10	100%
	• Sufficiently specific and comprehensive material	10	100%

## Discussion

The development and validation of the MILD EduB addresses a critical gap in patient education for individuals with ILD in India. Unlike patients with COPD, whose educational resources are well established, ILD-specific materials are scarce, particularly in low- and middle-income countries, where cultural and linguistic barriers further limit accessibility [38]. MILD EduB was designed to overcome these challenges by providing a structured, evidence-based, and culturally adapted resource tailored to the needs of Indian patients. With thorough enhancements, the MILD EduB was able to meet rigorous content validity standards, indicating its effectiveness and reliability as a patient education tool.

Our findings demonstrate that the MILD EduB has high content validity, with an initial S-CVI/Ave of 0.79, which improved to 1.0 after expert revisions. This rigorous validation process aligns with methodologies used in other chronic respiratory disease interventions, such as those for COPD [40]. However, while COPD education programs often focus on smoking cessation and airway clearance, MILD EduB incorporates ILD-specific themes, including pathophysiology of ILD, clinical tests for ILD, managing breathlessness/dyspnea, managing cough, managing flare-ups, prognosis, managing anxiety, panic, and depression, using supplemental oxygen, using nocturnal oxygen, regular vaccination, managing medications and side effects, good nutrition, managing coexisting medical conditions, accessing home-care and support for patients and caregivers, quitting smoking, symptoms of gastroesophageal reflux disorder, adherence to medications, life-style changes, exercise, end-of-life care and advanced directives, benefits of pulmonary rehabilitation, importance of exercise, and taking control of their own health; the topics rarely emphasized in generic pulmonary rehabilitation materials [11].

A key strength of MILD EduB is its patient-centered design, reflected in the unanimous approval (100% agreement) from ILD participants regarding readability, relevance, and usability. The final revised version of booklet’s high Flesch Reading Ease score (80.9) further supports its suitability for diverse literacy levels, a feature often lacking in Western-developed resources [37,38].

The multidisciplinary approach used in developing the MILD EduB incorporates input from pulmonologists, physiotherapists, and patients which mirrors best practices in chronic disease education. Futhermore, our work specifically targets people with ILD, a disease with distinct pathophysiology and management challenges. Unlike COPD-focused education, which emphasizes airway clearance and bronchodilator use, ILD education requires greater emphasis on oxygen therapy, energy conservation, and exercise monitoring due to its restrictive pathophysiology [11].

PR includes a variety of interventions that are centered around the patient’s specific needs, with PE an important component of PR [41]. PE has been proven to help patients develop self-management skills while improving long-term adherence to exercise and changes in their lifestyle through providing patients with information that is essential for the long-term management of their conditions [41]. The goal of educational initiatives is to improve disease management and enhance self-efficacy. The importance of PEs in treating chronic lung diseases is emphasized in several guidelines issued by national and international advisory groups [1, 20, 42,43]. Good health education aims to improve the lives of individuals by promoting positive changes in attitudes, values, awareness, abilities, and behavior [44]. To deliver effective, cost-efficient, and high-quality health education, the educational material should be easily accessible and strategically focused. Educational programs for individuals with ILD cover a variety of topics, including oxygen therapy, energy conservation, managing symptoms, improving mental health, medications, lung transplantation, and palliative care. In addition to receiving individualized care, patients can learn more about their condition, diagnosis, symptoms, drugs, coping mechanisms, and pulmonary rehabilitation [19].

The developed and validated MILD EduB educational-exercise booklet can be used as an educational resource to help fill the educational component of PR for individuals with ILD. It could potentially improve adherence to PR. To effectively manage the course of the disease, treating people with ILD requires a multidisciplinary approach, requiring the collaboration of healthcare staff, patients, and caretakers. A multidisciplinary group of respiratory physicians, physiotherapists and individuals with ILDs contributed to the process of evaluating the educational booklet’s content and presentation.

To reduce the increasing burden of chronic diseases and empower patients to take a more active part in their own care, PE is essential [45]. Patients can readily read and revisit the information in the educational booklet at any time, making it a cost-effective and convenient resource. It is a flexible and potentially helpful tool to address the increasing prevalence of chronic diseases in society [21–25,45]. In accordance with our findings, participants (individuals with ILD) requested that exercise training recommendations be included in the educational booklet. Despite an increase in educational resources for chronic conditions, there have been no documented booklets explicitly for individuals with ILD in India. Our study presents ‘MILD EduB’, an illustrated educational booklet that is self-explanatory and intended to assist individuals with ILD in managing their disease and performing exercise on their own. Upon receipt of the booklet, respondents felt that they had become more knowledgeable about the disease. Such findings suggest that the booklet may facilitate self-management in the future. However, further evaluation is needed to assess the effects on patient behaviors, compliance, and longer-term clinical outcomes.

Despite these advancements, some limitations must be acknowledged. The current version of the MILD EduB is available only in English, which may restrict its use among non-English-speaking populations. Although caregivers play a critical role in ILD management, they were not directly included in the booklet validation process, which should be addressed in future iterations. Future studies should focus on translating and validating the booklet in regional languages to increase accessibility. Additionally, while initial feedback from patients and healthcare professionals is promising, long-term studies are needed to assess the impact of the booklet on clinical outcomes, such as hospitalizations, disease progression, and quality of life. Given that public health care employees might be less familiar with ILD, healthcare provider training is crucial. Adoption would be enhanced by creating short training sessions on booklet usage and ILD management for community health workers. Generalizability is limited by the small patient validation sample, although it is adequate for first-person validation. To evaluate wider acceptance, future research should involve a larger, geographically varied group. The absence of long-term impact data on clinical outcomes (such as hospitalization rates and quality of life) remains an area of concern. To assess practical effectiveness, a multicenter RCT comparing MILD EduB users to standard care could be undertaken. Access to online copies of the booklet may be impeded in rural regions because of technological disparity. This gap might be filled by a hybrid distribution strategy that provides video-based versions of the existing booklet.

## Conclusion

MILD EduB is a significant advancement in ILD education, especially considering resource limited environments such as India. Through combining culturally adapted evidence-based content, Phase 1 fills an important gap in development of patient centered educational tools for individuals with ILD. Demonstrated herein are that both healthcare providers and patients find MILD EduB relevant, readable and acceptable. Nevertheless, its influence on patient knowledge, adherence, self-management and clinical outcomes remain to be evaluated in a subsequent randomized controlled trial.

## Abbreviations

COPD: Chronic Obstructive Pulmonary Disease
CRDs: Chronic Respiratory Diseases
CTRI: Clinical Trials Registry of India
CTD-associated ILD: Connective Tissue Disease-associated ILD
CONSORT: Consolidated Standards of Reporting Trials
CVI: Content Validity Index
HP: Hypersensitivity Pneumonitis
ILD: Interstitial Lung Disease
I-CVI: Item-Level Content Validity Index
KMC and KH: Kasturba Medical College and Kasturba Hospital
MILD EduB: Manipal Interstitial Lung Disease Educational Booklet
PE: Patient Education
QoL: Quality of Life
RCT: Randomized Controlled Trial
S-CVI/Ave: Scale-Level Content Validity Index
